# A real-world retrospective study of the use of Ki-67 testing and treatment patterns in patients with HR+, HER2− early breast cancer in the United States

**DOI:** 10.1186/s12885-022-09557-6

**Published:** 2022-05-06

**Authors:** Jacqueline Brown, Savannah Scardo, Michael Method, Dan Schlauch, Amanda Misch, Shaita Picard, Erika Hamilton, Suzanne Jones, Howard Burris, David Spigel

**Affiliations:** 1 Eli Lilly and Company Limited, 8 Arlington Square West, Downshire Way, Bracknell, RG12 1PU UK; 2 Eli Lilly and Company, Indianapolis, IN USA; 3 Genospace, Boston, MA USA; 4 Sarah Cannon, Nashville, TN USA; 5 Tennessee Oncology, Nashville, TN USA

**Keywords:** Breast neoplasm, Early breast cancer, Diagnostic test, HR+ breast cancer, Ki-67, Ki-67 index, Retrospective study, Treatment pattern

## Abstract

**Background:**

The National Comprehensive Cancer Network recommends that patients with hormone receptor-positive early breast cancer be considered for adjuvant endocrine therapy (ET) after primary treatment like surgical excision. Adjuvant chemotherapy (CT) use primarily depends on risk of recurrence. Biomarkers such as Ki-67 potentially have most value in patients with intermediate risk factors, such as involvement of 1–3 positive nodes. This study evaluated the use of Ki-67 testing and treatment patterns in patients with HR+, human epidermal growth factor receptor 2-negative early breast cancer.

**Methods:**

This was an observational retrospective cohort study of patients with electronic medical records from January 2010 to August 2018 treated for HR+, HER2− early breast cancer at Sarah Cannon sites in the United States (US). Overall, 567 patients were randomly selected after using the eligibility criteria: female or male ≥18 years, without distant metastases, and with available physician and pathology reports. Multivariable logistic regression was used to investigate factors predicting Ki-67 testing and test results. Descriptive analyses were applied to treatment patterns.

**Results:**

Multivariable logistic regression analyses found no clinical or pathological factors that predicted whether Ki-67 testing had been ordered by physicians. Of all tested patients (*N =* 130), having Grade-2 tumors (OR, 7.95 [95% CI: 2.05, 30.9]; *p* = 0.0027) or Grade-3 tumors (OR, 95.3 [95% CI, 11.9, 760.7]; *p* < 0.001) at initial diagnosis was a predictor of high Ki-67 expression (≥20%). Ki-67 expression was tested in 23.6% (61/258) of patients with 1–3 positive nodes; 54.1% of them (33/61) had high Ki-67 expression (≥20%). While having a higher grade tumor predicted high Ki-67 (≥20%), 28.6% of patients with Grade-1 tumors also had high Ki-67 expression. Neo-adjuvant therapy was received by 16.0% of patients (91/567), most of whom (66/91; 72.5%) received CT alone. Adjuvant therapy, either endocrine and/or chemotherapy, was received by 92.6% (525/567) of patients and by 67.0% (61/91) of those who received neo-adjuvant therapy. Most (428/525, 81.5%) received ET in the adjuvant treatment setting.

**Conclusions:**

High grade tumors predicted high Ki-67 (≥20%) expression, but Ki-67 testing was not widely used in these US patients. Most HR+, HER2− early breast cancers were treated with adjuvant ET, with or without CT.

**Supplementary Information:**

The online version contains supplementary material available at 10.1186/s12885-022-09557-6.

## Background

The most prevalent breast cancer subtype is hormone receptor-positive (HR+), human epidermal growth factor receptor 2-negative (HER2–), which accounts for around 70% of all breast cancers. Breast cancer is the second leading cause of cancer deaths after lung cancer among women in the United States (US) [1]. For 2021, 281,550 new cases of invasive breast cancer are projected to be diagnosed in the US, with approximately 43,600 dying from the disease [1].

In the US, most patients with breast cancer are diagnosed with early-stage disease. These patients are candidates for local treatments with curative intent, such as surgery followed by radiotherapy, depending on the surgical approach and nodal involvement. Patients may also receive some form of chemotherapy (CT), either before (neo-adjuvant) or after (adjuvant) surgery, especially those with high risk of recurrence, defined either by clinical pathologic factors (eg, tumor grade, size, nodal status), multi-gene assay, or other biomarkers. For estrogen receptor (ER)-positive or progesterone receptor (PR)-positive tumors, National Comprehensive Cancer Network (NCCN) Guidelines^1^ recommend that adjuvant endocrine therapy (ET) be considered regardless of the patient’s age, lymph node status, or whether adjuvant CT is administered [2]. The choice of endocrine agent, primarily tamoxifen or one of the three selective aromatase inhibitors, anastrozole, letrozole, or exemestane, is mainly driven by the patient’s menopausal status or the preferred side effect profile of the agents.

Despite the fact that most early-stage HR+ patients receive adjuvant ET with curative intent, approximately 30% go on to experience distant relapse with metastases [3]. There is currently no standard clinicopathologic definition predictive of high risk of disease recurrence. Almost all patients with ≥4 involved lymph nodes are considered high risk for recurrence and are typically treated with CT irrespective of other factors. Patients with 1–3 involved lymph nodes have a risk of recurrence that is more dependent on additional factors, such as large primary tumor size, high histologic grade as defined by the Nottingham Grading System, and, most recently, the results of multi-gene assays [4].

Tumor proliferation has been considered an important prognostic biomarker to determine risk in early breast cancer [2, 5, 6]. Proliferation is often determined through measuring Ki-67 antigen, a nuclear protein expressed in all phases of the cell cycle except the G0 phase [7]. High tumoral Ki-67 levels are associated with higher risk of recurrence [8, 9]. Ki-67 testing by immunohistochemistry is accessible, relatively inexpensive, and easy to perform [10].

Ki-67 can be used as a predictor of prognosis in HR+, HER2− breast cancer and, together with low PR expression, it may be particularly useful for discriminating luminal A from higher risk luminal B cases [7, 11, 12]. A retrospective study of HR+, HER2− breast cancer patients in Germany was able to differentiate patients’ disease-free survival according to the Ki-67 expression levels. The 5-year probability of disease-free survival was 0.90 for patients with low Ki-67 levels (< 10%), 0.89 for intermediate levels (10 to 19%), and 0.77 for high levels (≥20%) [11]. Ki-67 was also able to further differentiate patients with an intermediate prognosis into different prognostic groups relative to other clinical parameters such as age, tumor grade, and disease stage. In another retrospective series of ER+, HER2− breast cancer patients, integrating Ki-67 index into the American Joint Committee on Cancer (AJCC) 8th Edition’s prognostic staging system helped identify patients with good prognosis, for whom treatment de-escalation could be considered [12]. In the recent monarchE Phase 3 randomized trial, high levels of Ki-67 (≥20%) in patients with 1–3 positive nodes were used to select patients with high-risk ER+, HER2− breast cancer for ET with or without abemaciclib and inclusion in the study [13]. The monarchE study met its primary end point of invasive disease-free survival in the intent-to-treat population [13].

The Ki-67 ≥ 20% threshold used in monarchE was based on the St Gallen International Expert Consensus on the Primary Therapy of Early Breast Cancer 2015-accepted Ki-67 levels of 10 to 20% to indicate an intermediate risk group (interpreted in the light of local laboratory values) and 20 to 29% to indicate a higher risk “luminal B-like” disease [14], which may be appropriate for adjuvant CT. However, international committees generally do not recommend Ki-67 testing in routine practice due to lack of standardization and assessment method reproducibility [15]. More specifically, the NCCN and American Society of Clinical Oncology currently do not recommend that treatment decisions be based on Ki-67 assessment [2, 16].

Real-world Ki-67 testing use is not well characterized among patients with HR+, HER2− early-stage breast cancer. The objectives of this study were to evaluate the distribution of Ki-67 testing status (yes vs no) and Ki-67 expression status (< 20% vs ≥20%) in patients with HR+, HER2− early breast cancer, highlighting patients with 1–3 involved lymph nodes, as well as the patterns of treatment in these patients.

## Methods

### Study design

This was an observational, retrospective cohort study of patients with early-stage HR+, HER2− breast cancer treated at Sarah Cannon HCA Healthcare sites in the US from January 1, 2010, to August 31, 2018. This time period was chosen because HER2 status and HER2-inhibitor treatment reporting became a requirement in 2010, and August 31, 2018 was the data cut-off for the study. Data were obtained from Genospace, a Web-based clinical trial matching and data aggregation/analysis platform that manages the Sarah Cannon electronic medical record (EMR) database. Three main clinical sites in the US, situated in Tennessee, Florida, and Colorado (each made up of multiple individual clinics), are represented in the Sarah Cannon database. The EMRs include structured medical fields, patient notes, and pathology reports including genomic (Ki-67) data. Many of the critical fields were captured primarily in unstructured sections of pathology reports and physician notes, requiring manual abstraction. The study was approved by the Institutional Review board of Sarah Cannon, the Cancer Institute of HCA Healthcare (Sarah Cannon Outcomes Master Retrospective Protocol [MR01]). Research was performed in accordance with the Declaration of Helsinki. Informed consent was not required because the patient data were de-identified before receipt.

### Analysis population

For the period January 1, 2010, to August 31, 2018, EMRs were available from 71,130 patients diagnosed with breast cancer. A representative sample of patients was selected at random using the ‘R’ programming language from the patients meeting inclusion and exclusion criteria. Random sampling was stratified by demographic characteristics (age, sex, race) to create a subset population closely matching the overall Sarah Cannon population. Additional patients were randomly sampled to replace those excluded. Female or male patients were included if they were aged ≥18 years, initially diagnosed with HR+, HER2− invasive early breast cancer (node-positive or -negative) without evidence of distant metastases, and with physician and pathology reports available. HR+, HER2− early breast cancer was defined as invasive breast cancer, Stage I–IIIC, and included regionally advanced (IIIB–C) disease [13]. This definition of EBC is based on the fact that treatment of EBC is with curative intent, as opposed to the metastatic (Stage IV) palliative care setting (NCCN Guidelines). In addition, patients were excluded if they had a prior history of breast cancer, evidence of any other primary malignancy (except non-melanoma skin cancer or other benign in situ neoplasm), or had received ET for breast cancer prevention (ie, tamoxifen, raloxifene, or aromatase inhibitors with no diagnosis of breast cancer).

Data included patient demographics and tumor characteristics (including tumor grade; stage; ER, PR, and HER2 status; and number of positive lymph nodes) at initial diagnosis, Ki-67 test status (yes vs no) and Ki-67 expression status (< 20% vs ≥20%), family history of breast cancer, type of insurance, and anti-cancer treatments administered. If not stated in patient notes, menopausal status was determined as being aged ≥60 years or < 60 years, with bi-lateral oophorectomy in line with the NCCN definition. Ki-67 data were abstracted from unstructured data sources including physician notes and pathology reports. All patients were assumed to have had their tumors resected. Treatments were assumed to be neo-adjuvant if given prior to surgery and adjuvant if given after surgery.

### Statistical analysis

Descriptive statistics were used to summarize patient, tumor, and Ki-67 testing characteristics in the overall cohort and the sub-cohort with pathological tumor involvement in 1–3 ipsilateral axillary nodes. Neo-adjuvant and adjuvant therapies were presented in chronological order, as documented in the abstracted data. If a treatment was documented in a patient’s record, it was assumed that the treatment had been received by the patient. No distinction was made between a patient receiving different therapies in combination or in succession. Neo-adjuvant and adjuvant treatments were summarized descriptively for the overall cohort and grouped according to receipt of ET and/or CT. Multivariable logistic regression models were run for the overall cohort using the following variables to identify marginal associations with both Ki-67 testing status (yes vs no) and Ki-67 expression status (< 20% vs ≥20%): age (measured as a continuous variable), insurance status (categorized as commercial, government, both, or none), family history of disease (categorized by primary and secondary family), date of diagnosis (measured as a continuous variable), tumor grade at initial diagnosis (Grade 1, 2, 3, or 4), number of nodes resected (measured as a discrete variable), nodes (0 or ≥ 1 nodes), tumor size (measured as a continuous variable), and histology (categorized as mammary, ductal, lobular, medullary not otherwise specified, mucinous, papillary, tubular, Paget’s, squamous cell, cribriform). The multivariable logistic regression analyses were run for the overall cohort to identify clinical or pathological factors that 1) predicted whether patients were tested for Ki-67 and 2) predicted high Ki-67 (≥20%) among those tested. Patients who were not tested for Ki-67 were removed from the Ki-67 expression status analyses. Histological data were removed for patients with fewer than five instances of a specific histology. Multivariable logistic regression analyses were performed using the ‘glm’ package in ‘R’. If data for the following fields were not referenced anywhere in the medical records, it was assumed that the record/test did not occur: family history of breast cancer; therapies; Ki-67 testing; and ER, PR, and HER2 testing. In addition, for the following fields, missing data were treated as missing completely at random for the purposes of statistical analysis: sex, age, race/ethnicity, tumor stage, grade, size, nodal status, histology, menopausal status, and Eastern Cooperative Oncology Group (ECOG) performance status.

## Results

### Patient characteristics

The study included 567 randomly selected patients who met the inclusion criteria (Table 1). All subjects were females, reflecting the low prevalence of breast cancer in male patients. The overall mean age was 61.8 years (SD = 12.7). Most patients were Caucasian (72.7%) and postmenopausal (79.2%). Approximately two-thirds of patients (65.3%) were originally diagnosed with Stage II cancer. Most tumors were 0–2 cm (63.1%) and Grade 1 (in 33.3% of patients) or Grade 2 (47.1%). Around half of the patients (45.5%) had pathological tumor involvement at 1–3 ipsilateral axillary lymph nodes, 37.4% had no lymph node involvement, and 17.1% had involvement of ≥4 nodes.

**Table 1:** Demographics and clinical characteristics of patients with HR+, HER2− early breast cancer at initial diagnosis

Characteristic	Overall	Node-Negative			1–3 Positive Nodes			≥4 Positive Nodes
		All	Tested for Ki-67	Not Tested for Ki-67	All	Tested for Ki-67	Not Tested for Ki-67	
N	*N =* 567	*N =* 212	*N =* 48	*N =* 164	*N =* 258	*N =* 61	*N =* 197	*N =* 97
Sex, n (%)								
Female	567 (100.0)	212 (100.0)	48 (100.0)	164 (100.0)	258 (100.0)	61 (100.0)	197 (100.0)	97 (100.0)
Mean age^a^ [SD], years	61.8 [12.7]	64.1 [12.4]	65.2 [12.0]	63.7 [12.5]	60.9 [12.8]	61.8 [12.1]	60.6 [13.0]	59.2 [12.1]
Race/Ethnicity^b^, n (%)								
American Indian or Alaska Native	1 (0.2)	1 (0.5)	0 (0.0)	1 (0.6)	0 (0.0)	0 (0.0)	0 (0.0)	0 (0.0)
Asian	3 (0.5)	1 (0.5)	0 (0.0)	1 (0.6)	2 (0.8)	0 (0.0)	2 (1.0)	0 (0.0)
Black or African American	35 (6.2)	10 (4.7)	2 (4.2)	8 (4.9)	15 (5.8)	3 (4.9)	12 (6.1)	10 (10.3)
Hispanic or Latino	21 (3.7)	9 (4.2)	4 (8.3)	5 (3.0)	6 (2.3)	1 (1.6)	5 (2.5)	6 (6.2)
Native Hawaiian or other Pacific Islander	2 (0.4)	2 (0.9)	0 (0.0)	2 (1.2)	0 (0.0)	0 (0.0)	0 (0.0)	0 (0.0)
White or Caucasian	396 (69.8)	147 (69.3)	32 (66.7)	115 (70.1)	186 (72.1)	42 (68.9)	144 (73.1)	63 (64.9)
Other	212 (37.4)	76 (35.8)	19 (39.6)	57 (34.8)	99 (38.4)	22 (36.1)	77 (39.1)	37 (38.1)
Unknown/Unspecified	26 (4.6)	13 (6.2)	2 (4.2)	11 (6.7)	9 (3.5)	4 (6.5)	5 (2.5)	4 (4.1)
Menopausal status, n (%)								
Postmenopause	449 (79.2)	175 (82.5)	41 (85.4)	134 (81.7)	196 (76.0)	47 (77.0)	149 (75.6)	78 (80.4)
Premenopause	118 (20.8)	37 (17.5)	7 (14.6)	30 (18.3)	62 (24.0)	14 (23.0)	48 (24.4)	19 (19.6)
Stage of disease^c^, n (%)								
Stage I	56 (9.9)	27 (12.7)	9 (18.7)	18 (11.0)	29 (11.2)	11 (18.0)	18 (9.1)	0 (0.0)
Stage II	370 (65.3)	164 (77.4)	35 (73.0)	129 (78.7)	191 (74.0)	45 (73.8)	146 (74.1)	15 (15.5)
Stage III	126 (22.2)	12 (5.6)	4 (8.3)	8 (4.8)	35 (13.6)	5 (8.2)	30 (15.2)	79 (81.5)
Unknown	15 (2.6)	9 (4.2)	0 (0.0)	9 (5.5)	3 (1.2)	0 (0.0)	3 (1.5)	3 (3.1)
Tumor size^c^, n (%)								
0–2 cm	358 (63.1)	105 (49.5)	24 (50.0)	81 (49.4)	195 (75.6)	43 (70.5)	152 (77.2)	58 (59.8)
> 2 to < 5 cm	155 (27.3)	83 (39.1)	20 (41.7)	63 (38.4)	48 (18.6)	15 (24.6)	33 (16.8)	24 (24.7)
≥ 5 cm	28 (4.9)	9 (4.2)	2 (4.2)	7 (4.3)	8 (3.1)	1 (1.6)	7 (3.6)	11 (1.3)
Missing	26 (4.6)	15 (7.1)	2 (4.2)	13 (7.9)	7 (2.7)	2 (3.3)	5 (2.5)	4 (4.1)
Number of positive nodes^c^, n (%)								
0	212 (37.4)	212 (100.0)	48 (100.0)	164 (100.0)	–	–	–	–
1	150 (26.5)	–	–	–	150 (58.1)	32 (52.5)	118 (59.9)	–
2	79 (13.9)	–	–	–	79 (30.6)	24 (39.3)	55 (27.9)	–
3	29 (5.1)	–	–	–	29 (11.2)	5 (8.2)	24 (12.2)	–
≥ 4	97 (17.1)	–	–	–	–	–	–	97 (100.0)
Histologic grade^c^, n (%)								
Grade 1	189 (33.3)	74 (34.9)	17 (35.4)	57 (34.8)	88 (34.1)	21 (34.4)	67 (34.0)	27 (27.8)
Grade 2	267 (47.1)	86 (40.6)	22 (45.8)	64 (39.0)	124 (48.1)	32 (52.5)	92 (46.7)	57 (58.8)
Grade 3	94 (16.6)	43 (20.3)	7 (14.6)	36 (22.0)	41 (15.9)	8 (13.1)	33 (16.8)	10 (10.3)
Unknown	17 (3.0)	9 (4.2)	2 (4.2)	7 (4.3)	5 (1.9)	0 (0.0)	5 (2.5)	3 (3.9)

*Abbreviation: SD* Standard deviation

^a^ One patient was excluded from the calculation due to an errant data point

^b^ Patients could select more than one category

^c^ Value at admission

### Ki-67 testing in overall cohort

**Fig. 1:**
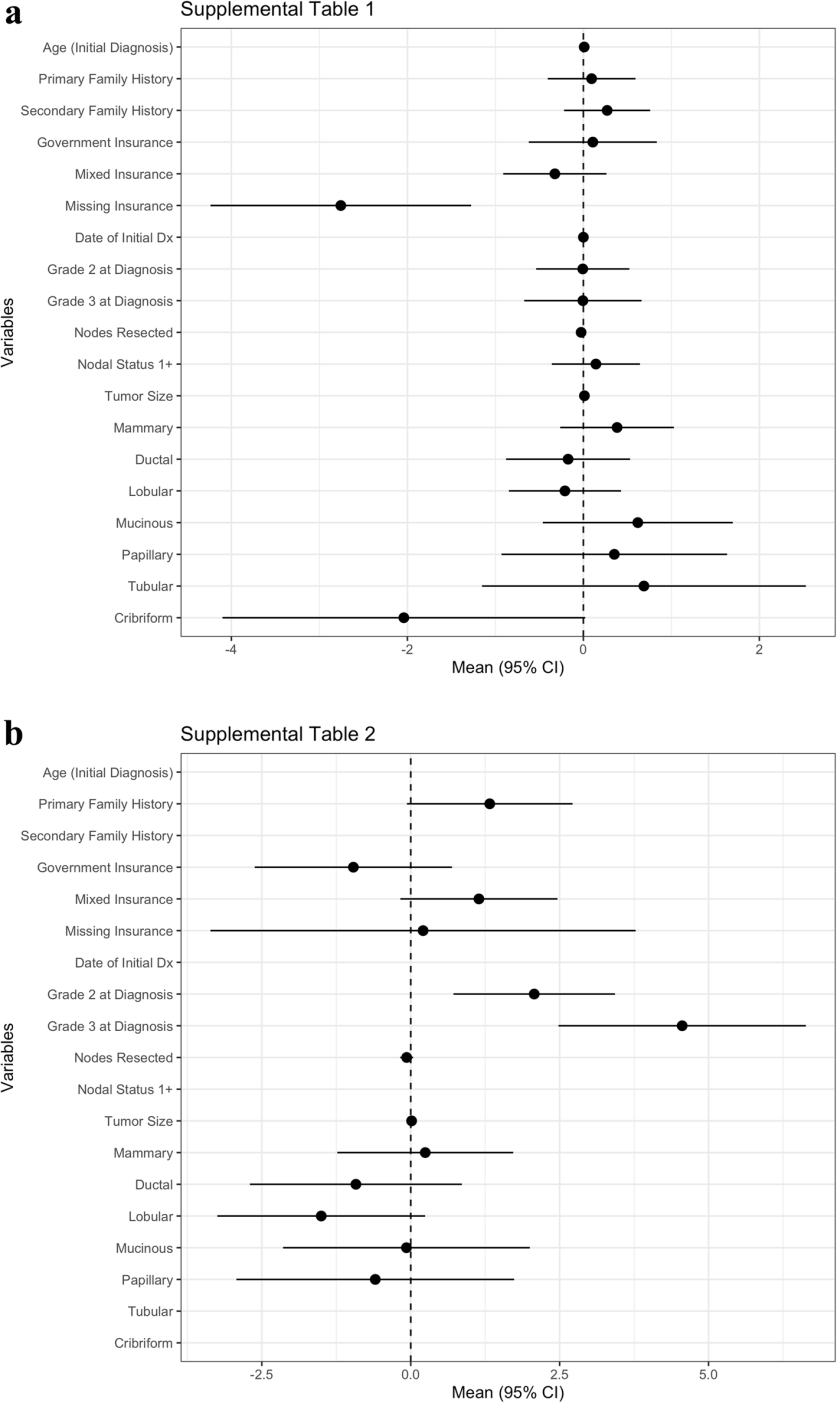
Multivariable logistic regression analyses. Forest plots showing the estimate values for the variables analyzed for association with (**a**) whether a patient was tested for Ki-67 (*N =* 567) and (**b**) a Ki-67 expression status ≥20% among those who were tested for Ki-67 (*N =* 130)

In total, 130 of the 567 patients (22.9%) were tested for tumor Ki-67 expression; 30 patients tested received neither neoadjuvant nor adjuvant treatment. Multivariable logistic regression of the total cohort (*N =* 567) showed that no clinical or pathological factors were predictors of whether a patient was tested for Ki-67 expression, although missing insurance was a negative predictor of testing (OR = 0.0635 [95% CI: (0.0144, 0.279)]; *p*-value = 0.0003; Fig. 1a). Of all patients tested (*N =* 130), more than half had high Ki-67 (≥20%; 74/130, 56.9%). Having Grade-2 tumors (OR = 7.95 [95% CI: 2.05, 30.9]; *p* = 0.0027) or Grade-3 tumors (OR = 95.3 [95% CI, 11.9, 760.7]; *p* < 0.001) at diagnosis was a predictor of high Ki-67 (≥20%; Fig. 1b).

### Ki-67 testing in patients with 1–3 positive nodes

Of the patients with 1–3 positive nodes, 23.6% (61/258) were tested for Ki-67 expression (Table 1). Those tested tended to have an earlier cancer stage at diagnosis: a greater proportion of Ki-67-tested patients had Stage I cancer than those not tested (18.0% vs 9.1%) and a lower proportion had Stage III cancer (8.2% vs 15.2%). Tested patients also more frequently had tumors > 2 cm to < 5 cm in size than those not tested (24.6% vs 16.8%), two positive nodes (39.3% vs 27.9%), and Grade-2 tumors (52.5% vs 46.7%; Table 1).

Of the Ki-67-tested patients with 1–3 positive nodes, 54.1% (33/61) had high Ki-67 (≥20%; Table 2). High Ki-67 was common among patients with 1–3 positive nodes who had Grade-2 (59.4%) or Grade-3 (100.0%) tumors, although 28.6% of patients with Grade-1 tumors also had high Ki-67. Patients with a higher number of positive nodes were also more likely to have high Ki-67 (80.0% of those with three positive nodes, 62.5% with two, 43.8% with one).

**Table 2:** Characteristics of Ki-67-tested patients with 1–3 positive lymph nodes by Ki-67 expression status

Characteristic	Tested for Ki-67 (***N =*** 61)	Ki-67 Status expression status	
		Ki-67 ≥ 20% (***N =*** 33)	Ki-67 < 20% (***N =*** 28)
Mean age [SD], years	61.8 [12.1]	60.9 [12.6]	62.8 [11.6]
Race/Ethnicity^a^, n (% with characteristic)			
Black or African American	3 (4.9)	1 (3.0)	2 (7.1)
Hispanic or Latino	1 (1.6)	0 (0.0)	1 (3.6)
White or Caucasian	44 (72.1)	26 (78.8)	18 (64.3)
Other	3 (4.9)	1 (3.0)	2 (7.1)
Unknown/Unspecified	15 (24.6)	8 (24.2)	7 (25)
Menopausal status, n (% with characteristic)			
Postmenopause	47 (77.0)	26 (78.8)	21 (74.9)
Premenopause	14 (23.0)	7 (21.2)	7 (25)
Stage of disease^b^, n (% with characteristic)			
Stage I	11 (18.0)	5 (8.2)	6 (9.8)
Stage II	45 (73.8)	24 (72.7)	21 (74.9)
Stage III	5 (8.2)	4 (12.1)	1 (3.6)
Tumor size^b^, n (% with characteristic)			
0–2 cm	43 (70.5)	24 (72.7)	19 (67.9)
> 2 to < 5 cm	15 (24.6)	8 (24.2)	7 (25)
≥ 5 cm	1 (1.6)	0 (0.0)	1 (3.6)
Missing	2 (3.3)	1 (3.3)	1 (3.6)
Number of positive nodes^b^, n (% with characteristic)			
1	32 (52.5)	14 (42.4)	18 (64.3)
2	24 (39.3)	15 (45.5)	9 (32.1)
3	5 (8.2)	4 (12.1)	1 (3.6)
Tumor Grade^b^, n (% with characteristic)			
Grade 1	21 (34.4)	6 (18.2)	15 (53.6)
Grade 2	32 (52.5)	19 (57.6)	13 (46.4)
Grade 3	8 (13.1)	8 (24.2)	0 (0.0)

*Abbreviation: SD* Standard deviation

^a^ Patients could select more than one category, ^b^ Value at admission

### Treatment patterns

Almost all patients (97.9% [555/567]) received neo-adjuvant and/or adjuvant therapy, which could be either endocrine and/or CT. Neo-adjuvant therapy was received by 91 patients (16.0%); 72.5% of these patients received CT only and 27.5% received ET, either alone or in combination with CT (few exceptions noted in Table 3). Most patients (61/91; 67.0%) who received neo-adjuvant therapy went on to receive adjuvant therapy (Table 3). Totally, 82.0% of patients who went on to receive adjuvant therapy received CT only as neo-adjuvant therapy.

**Table 3:** Neo-adjuvant therapy

Neo-Adjuvant Therapy	Total (***N =*** 91)		No Subsequent Adjuvant Therapy (***N =*** 30)		Subsequent Adjuvant Therapy (***N =*** 61)	
	n	%	n	%	n	%
***Neo-adj CT only***^***a***^	***66***	***72.5***	***16***	***53.3***	***50***	***82.0***
Doxo–Cyclophos–Paclitaxel	31	34.1	7	23.3	24	39.3
Docetaxel–Doxo–Cyclophos	6	6.6	2	6.7	4	6.6
Doxo–Cyclophos–Docetaxel	6	6.6	3	10.0	3	4.9
Cyclophos–Doxo–Paclitaxel	3	3.3	0	0.0	3	4.9
Docetaxel–Cyclophos	3	3.3	1	3.3	2	3.3
Doxo–Cyclophos	3	3.3	1	3.3	2	3.3
Cyclophos–Doxo–Docetaxel	2	2.2	0	0.0	2	3.3
Other treatment	12	13.2	2	6.7	10	16.4
***Neo-adj ET only***^**b**^	***22***	***24.2***	***12***	***40.0***	***10***	***16.4***
Anastrozole	12	13.2	5	16.7	7	11.5
Letrozole	4	4.4	4	13.3	0	0.0
Palbociclib–Letrozole	2	2.2	1	3.3	1	1.6
Other treatment	4	4.4	2	6.7	2	3.3
***Neo-adj CT and ET***	***3***	***3.3***	***2***	***6.7***	***1***	***1.6***
Doxo–Cyclophos–Paclitaxel –Anastrozole	2	2.2	1	3.3	1	1.6
Doxo–Cyclophos–Paclitaxel –Letrozole	1	1.1	1	3.3	0	0.0

*Abbreviations: Neo-adj* Neo-adjuvant, *CT* Chemotherapy; cyclophos, cyclophosphamide; doxo, doxorubicin, *ET* Endocrine therapy

^a^ Includes a sequence that contained trastuzumab. ^b^ Includes sequences containing cyclin-dependent kinase 4 and 6 inhibitors (eg, palbociclib)

Adjuvant therapy was received by 525 patients (92.6%; Table 4). Most received ET in the adjuvant space (*n =* 428, 81.5%). Adjuvant treatments involving ET only (ie, without CT) were most common (47.4% [249/525]): single agent anastrozole was the most frequent adjuvant treatment sequence (23.4% [123/525]) followed by single-agent tamoxifen (8.0% [42/525]). Initial adjuvant treatment with an ET was used in a greater proportion of patients who had received neo-adjuvant treatment than those who had not (86.8% [53/61] vs 43.1% [200/464]).

**Table 4:** Adjuvant therapy

Adjuvant Therapy	Total (***N =*** 525)		No Prior Neo-Adj Therapy (***N =*** 464)		Prior Neo-Adj Therapy (***N =*** 61)	
	n	%	n	%	n	%
***Adj CT only***	***97***	***18.5***	***93***	***20.0***	***4***	***6.6***
Doxo–Cyclophos–Paclitaxel	38	7.2	38	8.2	0	0.0
Docetaxel–Cyclophos	26	5.0	24	5.2	2	3.3
Doxo–Cyclophos	11	2.1	11	2.4	0	0.0
Cyclophos–Docetaxel	5	1.0	5	1.1	0	0.0
Doxo–Cyclophos–Docetaxel	3	0.6	3	0.6	0	0.0
Cyclophos–Fluorouracil	3	0.6	2	0.4	1	1.6
Other treatment	11	2.1	10	2.2	1	1.6
***Adj CT followed by ET***^***a***^	***175***	***33.3***	***171***	***36.9***	***4***	***6.6***
Docetaxel–Cyclophos–Anastrozole	32	6.1	32	6.9	0	0.0
Doxo–Cyclophos–Paclitaxel–Anastrozole	25	4.8	25	5.4	0	0.0
Docetaxel–Cyclophos–Tamoxifen	15	2.9	15	3.2	0	0.0
Doxo–Cyclophos–Paclitaxel–Tamoxifen	10	1.9	10	2.2	0	0.0
Docetaxel–Cyclophos–Letrozole	9	1.7	8	1.7	1	1.6
Doxo–Cyclophos– Paclitaxel–Letrozole	6	1.1	6	1.3	0	0.0
Cyclophos–Docetaxel–Anastrozole	5	1.0	5	1.1	0	0.0
Doxo–Cyclophos–Paclitaxel–Tamoxifen –Anastrozole	4	0.8	4	0.9	0	0.0
Cyclophos–Fluorouracil–Anastrozole	3	0.6	3	0.6	0	0.0
Other treatment	66	12.6	63	13.6	3	4.9
***Adj ET only***^***b***^	***249***	***47.4***	***197***	***42.5***	***52***	***85.2***
Anastrozole	123	23.4	104	22.4	19	31.1
Tamoxifen	42	8.0	26	5.6	16	26.2
Letrozole	30	5.7	25	5.4	5	8.2
Anastrozole–Letrozole	13	2.5	11	2.4	2	3.3
Letrozole–Anastrozole	7	1.3	5	1.1	2	3.3
Anastrozole–Exemestane	6	1.1	4	0.9	2	3.3
Tamoxifen–Anastrozole	4	0.8	4	0.9	0	0.0
Exemestane	3	0.6	2	0.4	1	1.6
Letrozole–Exemestane	3	0.6	3	0.6	0	0.0
Other treatment	18	3.4	13	2.8	5	8.2
***Adj ET followed by CT***	***4***	***0.8***	***3***	***0.6***	***1***	***1.6***
Anastrozole–Paclitaxel	1	0.2	0	0.0	1	1.6
Anastrozole–Doxo–Cyclophos–Paclitaxel	1	0.2	1	0.2	0	0.0
Anastrozole–Letrozole–Exemestane –Docetaxel–Cyclophos–Tamoxifen	1	0.2	1	0.2	0	0.0
Letrozole–Cyclophos–Methotrexate –Fluorouracil–Tamoxifen	1	0.2	1	0.2	0	0.0

*Abbreviations: Adj* Adjuvant, *CT* Chemotherapy, *Cyclophos* Cyclophosphamide, *Doxo* Doxorubicin, *ET* Endocrine therapy

^a^ Includes sequences containing bevacizumab, enzalutamide, or palbociclib

^b^ Includes sequences containing cyclin-dependent kinase 4 and 6 inhibitors (eg, palbociclib or ribociclib)

Adjuvant treatments starting with CT followed by ET (33.3% [175/525]), or of CT alone (18.5% [97/525]), were also common.

More than half of the patients with 1-3 positive nodes who were tested for Ki-67 received CT (59.0% [36/61]): a higher percentage of those with high Ki-67 (≥20%) received CT compared to patients with low Ki-67 (< 20%); 66.7% vs 50.0%, respectively. Overall, receipt of a Ki-67 test did not appear to influence the treatment received (Table S1).

## Discussion

The decision to administer adjuvant CT and targeted agents is often complex in early HR+ breast cancers, especially among patients with 1–3 positive nodes who are often considered an intermediate risk group [10]. The benefit of such agents, particularly considering associated treatment-related toxicities, is debatable among patients with better prognosis. Although some have suggested Ki-67 testing can assist decision-making for, or against, adjuvant therapies in this population [6], clinical oncology guidelines do not currently recommend use in this way due to lack of standardization and assessment method reproducibility [2, 15, 16]. The use of Ki-67 testing in the adjuvant setting varies across regions and countries; for example, data from one study has shown that Europe conducted tests in 72% of patients, Japan tested 43% of patients, and the US conducted tests in 29% of patients [17]. In line with this, our study suggests that Ki-67 testing is not widely used in US patients with HR+, HER2− breast cancer, with less than a quarter (22.9%) of the patient population tested. The proportion of those tested was not much greater among patients with 1–3 positive nodes (23.6%), the population in which Ki-67 testing potentially has the most prognostic value.

Although no clinical or pathological factors in the multivariable analysis were associated with the Ki-67 test being performed, those that were tested were more likely to have T2 tumors (> 2 cm to < 5 cm), two positive nodes, and Grade-2 tumors. Patients without insurance were also less likely to get tested for Ki-67, suggesting financial reimbursement was a potential driver of use. High Ki-67 (≥20%) scores were not limited to high grade tumors (28.6% in Grade 1), but were more common among those with Grade-2 or Grade-3 tumors. Of patients who had 1–3 positive nodes and were tested for Ki-67, a higher percentage of those with high Ki-67 (≥20%) received CT compared to patients with Ki-67 < 20% (66.7% vs 50.0%, respectively). It is possible, but cannot be determined from the available data, that certain clinical sites or physicians routinely perform Ki-67 testing, whereas others do not.

Current treatment guidelines recommend ET as initial treatment, especially in postmenopausal women, with CT reserved for patients with high risk of recurrence [2, 5, 6]. Consistent with these guidelines, most patients (81.5%) who received adjuvant therapy were treated with ET, with or without CT. Single agent anastrozole was the most common adjuvant treatment, followed by the selective ER modulator tamoxifen (single agent). Third-generation aromatase inhibitors, such as anastrozole, letrozole, and exemestane, are the standard endocrine treatments in postmenopausal women in early-stage HR+ breast cancer [18]. In the adjuvant setting, 18.5% of patients received CT only; however, these patients may have gone on to receive ET beyond the last date of data extracted for inclusion in this study. Overall, a wide range of treatment sequences was used for treating early-stage HR+, HER2− disease despite the cancer centers belonging to the same health institution.

This study provides important real-world data on the extent of Ki-67 testing from a large patient cohort for whom the Ki-67 index has potential prognostic value. Limitations to the study include its basis on retrospective data, and that these data were mainly collected from three sites under the same institution, which may not be representative of the national population. Data fields within the database also varied in their availability and completeness and, for many of the critical fields, the human abstraction required from unstructured sections of pathology reports and physician notes could have introduced errors.

## Conclusions

The results from this study show that Ki-67 testing is not widely used in US patients with HR+, HER2− early breast cancer. Ki-67 score could become a useful prognostic marker to guide treatment decision-making in patients with HR+, HER2− early breast cancers with intermediate risk of disease recurrence. However, for it to be widely accepted, further research is needed to standardize Ki-67 measurement, optimize cut-off points for risk stratification, and confirm its clinical utility. In line with NCCN Guidelines, HR+, HER2− early breast cancers were mostly treated with ET with or without CT.

## Supplementary Information

**Additional file 1: Table S1** Neo-adjuvant and adjuvant therapies by Ki-67 testing status

## Data Availability

The data that support the findings of this study are available from Genospace, but restrictions apply to the availability of these data, which were used under license for the current study, and so are not publicly available. Data are, however, available from the authors upon reasonable request and with permission of Sarah Cannon, the Cancer Institute of HCA Healthcare.

## Abbreviations

AJCC: American Joint Committee on Cancer
CI: Confidence interval
CT: Chemotherapy
Cyclophos: Cyclophosphamide
Doxo: Doxorubicin
ECOG: Eastern Cooperative Oncology Group
EMR: Electronic medical record
ER: Estrogen receptor
ET: Endocrine therapy
HER2: Human epidermal growth factor receptor 2
HR: Hormone receptor
NCCN: National Comprehensive Cancer Network
Neo-adj: Neo-adjuvant
OR: Odds ratio
PR: Progesterone receptor
SD: Standard deviation

## Footnotes

1 National Comprehensive Cancer Network (NCCN) and NCCN are registered trademarks of NCCN
