# Supplementary material for: A real-world retrospective study of the use of Ki-67 testing and treatment patterns in patients with HR+, HER2− early breast cancer in the United States
Source: BMC Cancer. 2022 May 6;22:502. doi: 10.1186/s12885-022-09557-6 (PMC9074265; doi:10.1186/s12885-022-09557-6)
Supplement: Supplementary file 1 — Additional file 1: Table S1 Neo-adjuvant and adjuvant therapies by Ki-67 testing status [file 12885_2022_9557_MOESM1_ESM.docx]

# Supplemental Material

**Table S1. Neo-adjuvant and adjuvant therapies by Ki-67 testing status**

|  | **Total** | | **Ki-67 tested [initial diagnostic]** | | **Ki-67 Not-tested [initial diagnostic]** | |
| --- | --- | --- | --- | --- | --- | --- |
|  | **N=615 Unique patients N=555** | | **N=110 Unique patients N=100** | | **N=505 Unique patients N=455** | |
|  | **n** | **%** | **n** | **%** | **n** | **%** |
| **Neo-Adjuvant Therapy** | ***91*** | ***7.4*** | ***20*** | ***9.1*** | ***71*** | ***7.0*** |
| ***Neo-adj CT only*** | 3 | 0.2 | 0 | 0.0 | 3 | 0.3 |
| ***Neo-adj ET only*** | 66 | 5.4 | 8 | 3.6 | 58 | 5.7 |
| ***Neo-adj CT and ET*** | 22 | 1.8 | 12 | 5.5 | 10 | 1.0 |
| **Adjuvant Therapy** | ***525*** | ***42.6*** | ***90*** | ***40.9*** | ***435*** | ***43.0*** |
| ***Adj CT followed by ET*** | 175 | 14.2 | 29 | 13.2 | 146 | 14.4 |
| ***Adj CT only*** | 97 | 7.9 | 18 | 8.2 | 79 | 7.8 |
| ***Adj ET only*** | 249 | 20.2 | 42 | 19.1 | 207 | 20.5 |
| ***Adj ET followed by CT*** | 4 | 0.3 | 1 | 0.5 | 3 | 0.3 |
